# Triadic Perspectives on Decision Making in Psychiatry: A Qualitative Study on Service Users, Caregivers and Healthcare Professionals in Latvia

**DOI:** 10.3390/healthcare13121416

**Published:** 2025-06-13

**Authors:** Solvita Olsena, Inese Stars, Elza Eliza Rozenberga, Karina Konstantinova, Ksenija Baidina

**Affiliations:** Faculty of Medicine and Life Sciences, University of Latvia, LV-1004 Riga, Latviaelza.rozenbergs@gmail.com (E.E.R.); karinaokk@gmail.com (K.K.); k.baidina@inbox.lv (K.B.)

**Keywords:** decision making, psychiatry, service users, family caregivers, healthcare professionals, qualitative study, Latvia

## Abstract

Background/Objectives: Decision making is fundamental to personal autonomy and person-centered, rights-based psychiatric care. This study explored the perceptions and experiences of decision making in psychiatric care from the perspectives of service users, caregivers, and healthcare professionals, identifying contexts that facilitate or hinder these practices. Methods: An exploratory–descriptive qualitative design was applied, using semi-structured interviews with eight service users, six caregivers, and seven healthcare professionals (21 interviews in total). Data were transcribed verbatim and analyzed through inductive content analysis. Results: Four main categories were identified: (1) antecedents for decision making, (2) challenges in decision making, (3) consequences of decision making, and (4) recommendations for improvement. Conclusions: This study provides insight into how decisions in psychiatric care are made in Latvia, highlighting the legal, personal, and institutional factors influencing these processes. Participants offered valuable perspectives, identifying both facilitators and barriers to autonomous decision making, as well as suggesting necessary improvements. The findings suggest the need for legal reform and systemic improvements in practices that favor agency in decision making. Additionally, they underscore the importance of strengthening individual capacities to support meaningful collaboration among service users, caregivers, and healthcare professionals in making healthcare decisions that enhance the quality of care and well-being of people with mental health difficulties.

## 1. Introduction

Decision making about one’s life—including the right to choose one’s mental healthcare—is fundamental to personal autonomy and personhood [1]. International human rights laws and medical ethics codes, including the Convention on the Rights of Persons with Disabilities (CRPD) [2], and national laws such as the Constitution of Latvia [3] and the Law on the Rights of Patients of Latvia [4] protect the dignity and integrity of individuals in mental healthcare. These laws also require the protection of informed consent rights. However, individuals with cognitive or psychosocial disabilities have historically been—and continue to be—disproportionately affected by substitute decision-making regimes [5].

The decision-making process is complex and multifaceted for many reasons, involving several key stakeholders—i.e., service users, family members, and healthcare professionals—each of whom contributes based on their distinct roles, responsibilities, and viewpoints. (In this article, we have chosen to refer to people who use mental health services as ‘service users’ rather than ‘patients’. In choosing this term, we have followed the recommendations of the European Network of (Ex-)Users and Survivors of Psychiatry. In Latvia, the definition of ‘patient’ is contained in Article 1(11) of the Law on Medical Treatment [6], which states that a patient is a person who receives or seeks healthcare services.) This process is shaped by a variety of interrelated factors, which may either support or hinder its effectiveness. These include institutional practices related to legal and ethical standards, cultural norms, the health status of service users, the availability of support systems, the attitudes and competencies of professionals, applicable legal frameworks, and broader resource constraints. Moreover, qualitative research consistently demonstrates that patients, their relatives, and clinical staff often perceive the decision-making process in a variety of different ways. These divergences are largely rooted in differing personal experiences, informational access, and expectations between the groups involved [7,8,9,10,11].

Research often focuses on the perspectives of either healthcare professionals [12,13,14], service users or patients [15,16], or other stakeholders, such as family members, caregivers in social services, and peer support workers [17,18,19,20]. However, these perspectives are typically examined in isolation. Some studies have adopted a dual-perspective approach, involving people with mental illnesses and their caregivers [9,21] or healthcare professionals [22]. The use of a triadic perspective in studies is less common but highly valuable [23,24,25].

The exploration of triadic perspectives in decision making is crucial because decisions are made collaboratively among service users, healthcare professionals, and caregivers. Studying only one or two perspectives in isolation cannot provide a complete understanding of the situation. By examining decision making in psychiatric healthcare from all major perspectives, the conclusions drawn can be more accurate and better reflect the reality of the situation. This will provide a more comprehensive understanding of decision making and care within mental health services.

Because of this, the current study employs a triadic perspective, building upon previous research that has incorporated multiple viewpoints [23].

Different types of decision making in mental healthcare are studied, ranging from substitute to shared and supported decision making [26]. These approaches reflect a spectrum of autonomy for service users, ranging from decisions made on their behalf to those made collaboratively or with support, depending on their decision-making capacity [26].

There is an increasing consensus on and strong advocacy for the implementation of a shared decision making (SDM) approach as an ethical imperative [27]. Several studies have shown that shared decision making (SDM) emphasizes the importance of therapeutic relationships in fostering better decisions [9,28]. The quality of the decision-making process enhances the overall care quality, while decision aids and SDM approaches improve treatment adherence and service user satisfaction [29].

According to the Convention on the Rights of Persons with Disabilities, Art. 12, supported decision making is required when a person with psychosocial disabilities experiences difficulties in making decisions [2]. Supported decision making enhances service users’ autonomy [28,30], empowering individuals to make choices about their lives while ensuring that these decisions are legally recognized. For healthcare professionals, supported decision making offers a clearer and more structured framework. Rather than relying solely on a binary assessment of capacity, i.e., whether someone can or cannot decide, it encourages a more flexible and collaborative process that supports the person in making their own decisions wherever possible. This process promotes better mental health outcomes, respects individual rights, and helps to reduce stigma surrounding cognitive impairments and paternalism [31]. Additionally, it decreases the application of coercive measures.

Recent studies on decision making in psychiatry indicate that greater service user involvement in and satisfaction with clinical decision making are associated with better outcomes [32]. Interventions such as coaching, peer support, and decision aids further enhance service user involvement [33]. Service users increasingly seek greater participation in treatment decisions and more balanced, collaborative relationships with psychiatrists [15].

There is a striking lack of data on patient decision-making processes in Latvian psychiatry. As a result, awareness of autonomy and the right to make free decisions, as well as the SDM approach in mental healthcare, is notably absent in our national context. To ensure that individuals with mental disorders have the right to make free and informed decisions in healthcare, it is crucial to collect and systematically assess data on the processes that either facilitate or hinder the realization of human rights.

The goal of the present study is to explore and describe the perspectives and practical experiences of service users, their caregivers, and psychiatric professionals regarding decision making in psychiatric care in Latvia. The study also seeks to examine the contextual factors that facilitate or hinder decision-making practices. The overall objective is to address the existing knowledge gap regarding this issue and to promote the implementation of a human rights-based approach in mental healthcare both nationally and worldwide.

Although international research provides valuable insights, it cannot fully capture the unique cultural, institutional, and legal specifics of the Latvian context. This study presents and discusses data from the legal, personal, and institutional domains in the Latvian context. It contributes to a deeper understanding of how decision-making processes in psychiatry are experienced by the three groups within the Latvian healthcare system. The findings underscore vital components of these processes and pinpoint prerequisites affecting their feasibility and quality. They emphasize the necessity of legal reforms and the implementation of systematic institutional improvements. It is hoped that this study will improve outcomes for service users and reduce barriers to realizing their rights in the decision-making process. Alongside international research and the current scientific literature, these findings will contribute to protecting the human rights of individuals with mental health challenges.

## 2. Materials and Methods

### 2.1. Study Design

This study employed an exploratory–descriptive qualitative (EDQ) research design, which is well suited for the investigation of understudied phenomena in healthcare. This approach provides a detailed description of the phenomenon of interest from the perspective of those who experience it [34].

### 2.2. Participants

The study included service users, their caregivers, and healthcare professionals. A purposive criterion sampling technique was employed to recruit participants based on predetermined inclusion criteria, focusing on informants with certain qualities or experiences relevant to research objectives [35]. The inclusion criteria for the participants are outlined in Table 1.

The recruitment of the study participants took place between February 2023 and August 2024 through official invitations distributed by healthcare professionals and online advertising. During this time, additional participants were identified via social networks, professional connections, and informal contacts with researchers. Individuals interested in participating reached out to the research team, who then provided details about the study and arranged interviews. Service users and family caregivers did not form dyads, i.e., they were unrelated individuals with no ties to each other.

This study involved 21 participants, including 8 service users, 6 family caregivers, and 7 healthcare professionals. The characteristics of the research participants are presented in Table 2. None of the participants requested to be withdrawn from the study. Information on potential participants who were approached but rejected the offer to participate was not collected or analyzed.

### 2.3. Data Collection

Semi-structured interviews were conducted to collect the data from May 2023 to August 2024. The research team developed three independent semi-structured interview guides, one for each group of study participants. The interviews’ guiding questions were based on the definition of supported decision making [36,37,38] and informed by a literature review on similar issues concerning service users [16,39], family caregivers [20,40,41], and healthcare professionals [42,43]. The interview questions were piloted before official interviews began to reduce bias. The topics covered in the interviews are summarized in Supplementary Table S1.

Research team members (K.K., K.B.) interviewed each participant individually. The interviewers did not know the participants before the study. A pre-meeting occurred only when an interviewer needed to meet an interviewee in person before the remote interview to obtain signed informed consent. Before the interviews, participants gave their informed written consent and self-identified as being able to participate fully. All participants were told that they could withdraw from the interview at any time for any reason. No financial compensation was offered for participation in the study.

The study participants chose the day, time, and location for the interviews. Ten interviews were conducted face to face, while 11 were conducted remotely. Remote interviews were conducted with three family members (caregivers), four service users, and four professionals. In-person interviews included three professionals, three family members, and four service users.

The interviews were audio-recorded with the participants’ permission. They lasted between 20 and 150 min and were transcribed verbatim in preparation for analysis. Personal data were protected throughout the study, and specific identifications were given to all participants (group ‘Service Users’: P1_1, P1_2, etc.; group ‘Family Caregivers’: P2_1, P2_2, etc.; group ‘Healthcare Professionals’: P3_1, P3_2, etc.).

### 2.4. Data Analysis

A qualitative content analysis was used to analyze the data based on Erlingsson and Brysiewicz’s (2017) data analysis process, which has an inductive orientation [44]. In the first stage, the reading and rereading of the transcribed interviews were performed to ensure familiarization with the data and achieve a general understanding of the participants’ perspectives. In the second stage, the interview text was divided into meaning units and then condensed to shorten them. The primary codes were determined in the third stage by assigning descriptive codes to the condensed meaning units. At the fourth stage of data analysis, similar primary codes were grouped into subcategories, which, in turn, were classified under more comprehensive categories with a higher level of abstraction to report the findings of the study. The data analysis steps were indicative, so the data analysis was carried out iteratively as a continuous rather than a linear process. Coding was performed manually.

The study was completed before saturation was achieved due to difficulties in recruiting participants (see the ‘Limitations’ section). To address this limitation, we focused on conducting a nuanced and transparent data analysis and reporting of the results, as suggested by Johnson et al. [45].

However, research rigor was ensured through triangulation and reflexivity. First, data source triangulation was employed, as data were collected from three perspectives: service users, caregivers, and health professionals. Second, two researchers (I.S., K.K.) independently coded and analyzed the data. One researcher (I.S.) held a PhD in Education Sciences and specialized in qualitative research, while the other (K.K.) was a PhD student and practicing psychiatrist. A third researcher (S.O.), who held a PhD in Medical Law and worked in patient advocacy, reviewed the assigned codes. Reflexivity was maintained through regular discussions among the research team throughout the data collection and analysis phases. Different perspectives were shared and considered during the interpretation of the data. Subcategories and categories were carefully discussed until a consensus was reached, resulting in the final set of subcategories and categories.

### 2.5. Ethics

This study received ethical approval from the Ethical Committee of the University of Latvia.

## 3. Results

**Figure 1:**
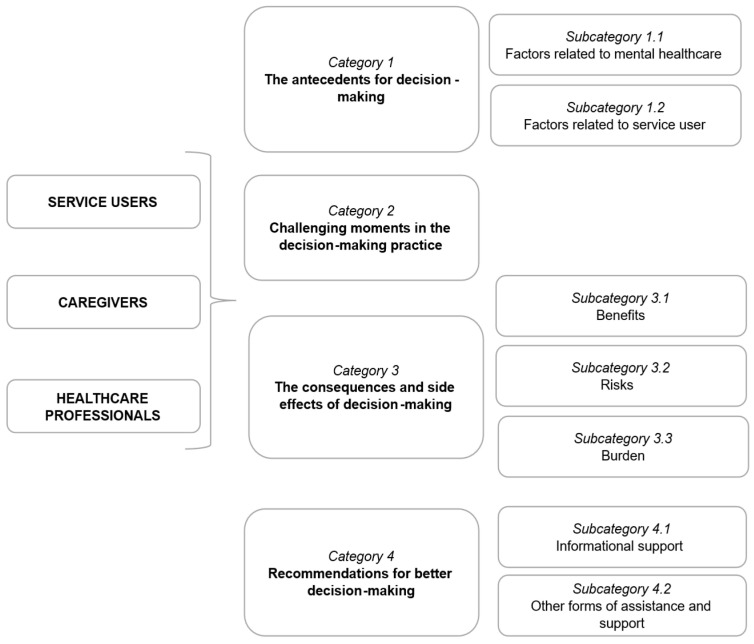
Map with categories and subcategories reflecting triadic perspectives: service users, caregivers, healthcare professionals.

The results are based on interviews with eight service users, six family caregivers, and seven healthcare professionals (HCPs). Figure 1 illustrates the categories and subcategories generated through the qualitative inductive content analysis, representing the participants’ experiences regarding decision making in psychiatric care. More detailed results, including the main codes, can be found in Table S2 of the Supplementary File.

### 3.1. Antecedents for Decision Making

The first category encompasses general circumstances that could influence the decision-making process from the participants’ perspectives.

#### 3.1.1. Factors Related to Mental Healthcare

This subcategory highlights various factors of mental healthcare that can either facilitate or hinder decision making about a service user’s health.

Service users

Above all, service users insisted that they must have regular and timely access to a psychiatrist to make decisions about their health. This was especially necessary during acute episodes. Both onsite and remote consultations using technology were recognized. The service users noted that regular communication with specialists allowed them to clarify issues and receive advice, which enabled them to make informed health decisions.

Furthermore, the appropriate duration of consultations was mentioned as a prerequisite for decision making. Service users expressed a desire for longer consultation times. Rushed and quick consultations caused additional stress and confusion for service users, limiting their ability to think calmly, ask questions about unclear issues, and reason before making a health decision.

Service users also mentioned several qualities of HCPs that were important for productive service user–doctor interactions and decision making. These included mutual trust and openness and empathetic, sensitive, tolerant, friendly, and non-critical attitudes, as well as an attitude free from arrogance and blame. Service users appreciated psychiatrists’ ability to listen to and hear, as well as to provide a conversation rather than a top-down monolog. They also highlighted the psychiatrist’s knowledge of the latest treatment options, ability to build a complete picture of the service user, responsiveness to their needs, and true interest in improving their quality of life.

Finally, service users described their expectations for truthful, accessible, clear, and understandable information about their mental health conditions and medical treatment options. Service users perceived the psychiatrist as the main source of this information. However, informational support was also received from internet sites, including social networks, books, and support groups. Service users felt that, to make a treatment decision, they should be informed about the treatment options. Moreover, they stressed that a lack of information can lead to incorrect decisions. Several service users emphasized the importance of assessing the reliability of the available information:

*“An informed decision is important to me. I am not going to take the place of a psychiatrist and decide what I will and will not take [medication], but it is important to me that I am informed about the medication and the options.”*

*[P1_3]*

Family caregivers

Several caregivers reported difficulties in obtaining accurate information about the service user’s health status due to a lack of communication with health professionals. These situations were more often experienced in hospitals than in outpatient settings. This made it difficult to make decisions about service user care, causing stress for caregivers. Caregivers stressed that if they are responsible for their service users, they should also have access to information about the service user’s health and treatment guidelines. Caregivers felt that it was the treating psychiatrist’s responsibility to provide them with this information discreetly, preferably during an onsite consultation.

Similarly, caregivers also emphasized service users’ right to be informed. They stated that healthcare professionals must inform service users about their health conditions, the treatment offered, the procedures to be performed, and the expected results. Service users should have the opportunity to express their views on the healthcare process. They have the right to be heard and to know their rights. Furthermore, information should be provided to the service user discreetly and without the presence of other individuals:

*“The patient must be fully informed. Thoroughly, nuanced, and deeply informed about everything happening to him/her. This is an elementary human right.”*

*[P2_3]*

Healthcare professionals

Healthcare professionals stressed that the rights of service users to participate in decision-making processes that affect their health should be accepted as a fundamental requirement in mental healthcare. HCPs believed that positive changes had already occurred in the healthcare system and practice, supporting and strengthening service users’ autonomy and decision making.

Regarding the information available to service users, the HCPs considered that access to information had already improved significantly. They believed that service users could obtain health information through various resources, such as leaflets, lectures, handouts, eHealth, and internet resources.

Additionally, several healthcare professionals felt that whether and to what extent a professional accepts service users’ participation in decision-making processes is, to some extent, related to the subjective views, beliefs, and character of the healthcare professional as an individual. The type of facility—outpatient or inpatient—can also have an impact. Some clinicians felt that service users’ autonomy may be less respected in hospitals.

#### 3.1.2. Factors Related to the Service User

This subcategory summarizes service user-related conditions that may affect their capacity to make healthcare decisions.

Service users

Service users highlighted their attitudes as a key component for productive interactions with healthcare professionals. They believe that service users should be cooperative, polite, interested, and self-initiating in their healthcare. Service users should not hide information about their health and symptoms from doctors and relatives.

Having a reliable support person was also mentioned as a factor that could influence decision making. All interviewed service users could name one or more people whom they classified as support persons and on whom they could rely in health-related situations. However, the psychiatrist was seen as the key person involved in health decisions. Some service users would trust the decisions about their health to other support persons (relatives, friends, colleagues) if necessary. In contrast, two service users reported that they did not wish to involve people other than professionals in their health decisions. They noted that a relative or friend could help the service user to travel to the doctor but not make decisions for them.

Service users perceived and experienced their mental health situations as a precondition that could facilitate or hinder decision making. Service users had noticed that the acute phase of the disease and severe symptoms (such as disinterest, drowsiness, euphoric feelings, overwhelming emotions, avoidance, being “in another reality” or “in a fog”) harmed their cognitive abilities and decision-making capacity. In the most severe episodes, they entrusted their decision making solely to the psychiatrist and, if necessary, to their support persons:

*“It doesn’t work for more serious episodes. I’ve also been on medication that slows down. Then no decision can be made. Just sleep and that’s it.”*

*[P1_2]*

*“At the moment of a manic episode, it is like being in another reality. It’s a different perception of reality and then it’s hard to make good decisions for yourself.”*

*[P1_4]*

Family caregivers

The service user’s ability to cooperate with clinicians and the caregiver was essential. However, caregivers faced challenging situations in which the service user was uncooperative and refused any help, including medication. The caregiver’s ability to influence the service user’s decisions was limited, leading to confusion about which decisions the caregiver should make regarding the service user’s healthcare.

Caregivers were convinced that the service user’s ability to perceive and evaluate the situation, as well as to make choices and decisions, was strongly influenced by the service user’s current mental health situation. Caregivers experienced that, in the acute and severe stages of the disease, the service user’s behavior was often unpredictable and inadequate. Additionally, the service user’s perception of reality was often affected by their medication. As a result, the service user was deemed to be unable to make appropriate healthcare decisions. Conversely, if the disease was well controlled and the service user’s mental health was good, he or she could be actively involved in the decision-making process regarding his or her healthcare. Furthermore, one mother felt that, due to her daughter’s complex diagnosis, she was unable to assess her daughter’s decision-making capacity.

Healthcare professionals

The HCPs were convinced that the service user must be interested in and motivated to participate in decision making, sufficiently active, and willing to collaborate with healthcare professionals. The service user was expected to take the initiative so that cooperation was reciprocal. The HCPs mentioned the concept of shared responsibility in this context. However, the HCPs highlighted that some health conditions can significantly affect a service user’s ability to be aware of their health status and limit their decision-making capacity (e.g., depression, affective bipolar disorder, severe cognitive impairment, acute psychotic states).

### 3.2. Challenging Moments in the Decision-Making Practice

The second category describes some critical moments experienced by service users, caregivers, and professionals in decision-making practices.

Service users

Decision making at the onset of the disease was noted as a challenge by four service users. The main factors that prevented service users from participating in their own health decisions were shame and confusion about the diagnosis, a fear of unfamiliar doctors, a lack of information about the disease, and a lack of experience with treatment. Here, one service user was afraid to disagree with the doctor and did not ask questions about aspects that they were worried about. Another service user pointed to insufficient information and no treatment experience at the onset of the illness, so more time was needed to decide, but this time was not always given.

Interestingly, service users concluded that they could make decisions more effectively about their health in an outpatient facility versus in a hospital. In outpatient consultations, the psychiatrist seemed to talk more with the service user, examine the problem more carefully, and offer different treatment options, giving the service user time to reflect. Outpatient psychiatrists seemed more accessible than inpatient psychiatrists.

Although all interviewed service users acknowledged that they needed information to make sound decisions about their mental health, a challenge was posed by the overwhelming amount of information and, at times, contradictory advice. This occurred when two or more physicians provided conflicting information to a service user on the same issue:

*“It is hard for me as a nonmedical person when two doctors tell me two different—opposite—things. I cannot make the right decision.” *

*[P1_6]*

Family caregivers

Regarding the onset of the service user’s illness, caregivers admitted that, at that time, they felt confused, worried, helpless, and even frightened by the significant changes in the service user’s behavior (e.g., symptoms). They also felt uncertain about the best course of action in this new situation. Caregivers noted that their decision to hospitalize a service user and admit them to a facility was emotionally difficult:

*“She [the daughter] started having hysterics. She was screaming and throwing herself on the ground. She did not go to the doctor. It only got worse. I remember it was difficult for me to call an ambulance the first time. It was difficult, emotionally it was difficult. It took me a while to call for help.”*

*[P2_2]*

Sometimes, this decision was accompanied by doubt, self-recrimination, guilt, and uncertainty about whether the caregiver had made the right decision.

Caregivers reported that, at certain stages of the illness, they were forced to take part in coercion or coercive-like behavior. Coercion had been mainly linked to the service user’s reluctance to be treated in the hospital during the acute and severe stages of the disease:

*“At the acute beginning of her illness, almost everything was imposed against her will. As parents, we insisted on her treatment because we saw that she was acting completely inadequate.”*

*[P2_1]*

These caregivers decided to hospitalize the service user involuntarily, either by themselves or through the emergency services, with or without the involvement of the police. Some caregivers had specific ‘strategies’ to achieve the desired service user behavior. For example, they used deception to persuade the service user to sign consent documents, or threats of hospitalization were used to force service users to take medications. Therefore, caregivers were forced to participate in coercive actions. This led to a variety of emotions, such as relief; feelings of victory, guilt, despair, anger, and pity; and doubts. However, caregivers reported that coercive tactics were used as the last option when all other methods failed, such as a verbal discussion about the need for treatment.

In some cases, caregivers and service users disagreed on what constituted coercion. For example, does verbal persuasion to go to the hospital constitute coercion? Does helping a service user to reach an ambulance (emergency medical transport) constitute coercion? These situations created conflicts between the caregiver and the service user.

In certain other cases, caregivers reported confusion about who made the main decisions about service users’ care and how this responsibility was shared. Is it a psychiatrist alone, a psychiatrist and a service user, a council of psychiatrists, or even a court? When and to what extent do caregivers become involved in this process? How significant is their influence? Caregivers reported that they needed clear guidance from HCPs on their level of responsibility to avoid ambiguous situations.

Several caregivers justified the use of coercion in specific situations. Coercion was justified when the service user endangered their own or others’ safety and health and when a medical consortium determined that coercive treatment followed the law:

*“When she is sick, things go so far that she stops taking her medication. She becomes hysterical, hits the dishes, and becomes aggressive and rude. Then the only thing left is to call an ambulance and take her to the hospital. And then comes treatment, including compulsory treatment by court order.”*

*[P2_2]*

*“This is clearly stated in the law. If a person is a danger to others, they must be treated and isolated from society. In this case, he/she must be treated forcibly.”*

*[P2_1]*

Healthcare professionals

Some psychiatrists felt as if they were caught between the service user and the caregiver in terms of the disclosure of information about service users’ health. In particular, psychiatrists reported uncomfortable situations when obtaining and sharing information about service users’ health with caregivers. For example, how much should they ask a family caregiver about a service user’s health, or how much about the service user’s health could they tell the service user’s caregiver? Professionals described suspicion among service users about so-called ‘withheld information’, where service users felt worried about what the caregiver told the psychiatrist or what the doctor told the caregiver. Such episodes made it difficult to build trusting relationships.

The service user’s relationship with the caregiver was another challenge. HCPs observed that the service user and caregiver relationship was not always positive and friendly. Decision making about the service user was more difficult if the service user was hostile toward the caregiver (or vice versa) or if there was mistrust and tension between them. Sometimes, affected by the disease, the service user had negative beliefs and fantasies about their caregiver. Doctors saw this as a difficulty because the service user did not trust either the doctor or the caregiver.

Difficulties in assessing a service user’s decision-making capacity were also noted by psychiatrists as a problematic issue. Healthcare professionals pointed out that there was no single structured tool to assess a service user’s decision-making capacity and to obtain relatively objective and consistent results. There were cases in which two specialists had different opinions about the same service user. Therefore, the psychiatrists concluded that it would be important to use a common tool. Currently, the evaluation of a service user’s decision-making capacity is based on clinical assessment. However, this assessment is also influenced by the physician’s experience, subjective views, and even personality traits.

Finally, two psychiatrists found that knowing that they had to make a decision about another person (in this case, the service user) was not easy. In particular, they found it difficult to come to the conclusion that they had to decide for the service user because their health condition did not allow it.

### 3.3. Consequences and Side Effects of Decision Making

This category summarizes information about the aspects that accompany the decision-making process from the perspectives of the three groups of participants.

#### 3.3.1. Benefits

This subcategory summarizes the positive aspects of the medical system, including the benefits of the current interaction between the three groups of participants.

Service users

The service users mentioned the benefits associated with medical treatment. They appreciated that the psychiatrist offered a choice of medications and gave them the option to choose. Service users highlighted that allowing them to contribute to the consultation process could lead to a more personalized pharmacological treatment plan. They insisted that the psychiatrist should listen to their views on medications, including side effects.

The service users felt safer and more secure when the psychiatrist talked to them, allowed them to express themselves, and offered treatment options. This led to a greater sense of control over their health situation and treatment.

The service users felt important when the psychiatrist listened; showed interest in their thoughts, feelings, and opinions; and gave them a voice. They appreciated being seen as partners in the treatment process.

Finally, two service users reported feeling empowered. They believed that the psychiatrist trusted their self-awareness and encouraged them to think for themselves and describe their symptoms, feelings, thoughts, actions, and beliefs—factors that can influence treatment.

Family caregivers

Caregivers felt that more active service user participation in treatment and care processes would increase service users’ autonomy and responsibility, which, in turn, would reduce their duties and burdens as caregivers.

Caregivers also assumed that if the service user made a specific decision about their health, they would be more likely to be motivated to implement it—for example, by taking their chosen medication or seeing their chosen psychologist.

Healthcare professionals

Healthcare professionals assumed that assessing a service user’s decision-making capacity allows for the more effective determination of their prognosis, treatment dynamics, service user cooperation, and treatment compliance.

In addition, the HCPs concluded that, even if a service user exhibits a significantly impaired decision-making capacity due to a medical condition, they can and should still be involved in simpler decisions, such as choosing food.

#### 3.3.2. Risks

Service users

Service users recognized that, during the acute stage of the disease, and while experiencing severe symptoms, they could make incorrect, dangerous, and harmful decisions about their health and safety.

Family caregivers

Caregivers acknowledged that deciding for someone else was a complex issue. According to caregivers, there are various risks of service user abuse, especially concerning involuntary treatment. There is also the risk that the caregiver’s intentions might be dishonest, selfish, or self-interested. Therefore, caregivers felt that the question of who makes the decision on behalf of the service user should be carefully considered in each service user’s situation.

Healthcare professionals

The HCPs expressed concern that the intentions of the service user’s caregiver were not always in the service user’s best interests. It was difficult, and sometimes even impossible, for the clinician to detect the caregiver’s hidden motives. For example, it is possible that the true interest of the caregiver may not be the health of the service user but their property or other material benefits. In another situation, the caregiver may have a personal dislike for the person whom they care for, which may also influence their decisions. Clinicians perceived this as a significant risk to the health and safety of service users.

#### 3.3.3. Burden

Service users

Two interviewed service users indicated that being involved in decision making was an additional burden, especially if they felt unwell. They also admitted that they had little interest in health topics, so they were reluctant to discuss the details of, for example, therapy. However, they had full trust in the physicians who treated them.

Family caregivers

Several caregivers pointed out that being responsible for the service user was an additional burden on their daily duties. Caregivers monitored the service user’s behavior to spot changes that could indicate a relapse. They cared for the service user daily and were involved in various decisions about the service user’s healthcare. Moreover, interactions with the service user were often complicated by symptoms of the service user’s illness, such as aggressive, defiant, or oppositional behavior; apathy; disinterest; unpredictable and manipulative behavior; denial of their illness; hiding or exaggerating symptoms; refusal to take medication, etc. Although caregivers tried to find and develop the most appropriate strategies to interact with service users, they described this as a difficult task.

Healthcare professionals

The professionals emphasized the necessity of additional resources to enhance the decision-making process. Firstly, the psychiatrist required more time to evaluate the service user’s decision-making capacity. Secondly, ensuring that service users have access to professional caregivers (as opposed to family caregivers) with a formal legal status and clearly defined responsibilities would entail recruiting and professionally training these individuals.

### 3.4. Recommendations for Better Decision Making

This category reflects ideas on how to make the decision-making process more inclusive and accessible to all stakeholders.

#### 3.4.1. Informational Support

Service users

Service users felt that they should receive more information about treatment plans and options, the risks associated with treatment and with not being treated, and how to deal with acute episodes. Service users should also be informed of their rights. This could be achieved through special brochures or other information tools (including digital ones) written in easy-to-understand language without complicated medical terminology. Some service users had already received such informational materials and appreciated them. The service users expressed their belief that, while in the hospital, they should be informed about the rules of the internal order of the hospital and the healthcare received, as well as leisure activities, clothing, and their diets. In addition, service users also needed more up-to-date information on other support options, such as support groups.

Family caregivers

Caregivers reported the need for more information in the form of specific recommendations, guidelines, formulas, or algorithms on how to deal with different situations related to the service user’s health and behavior. Caregivers also desired a list of things not to do when caring for a service user with a mental illness, to avoid unnecessary escalation. Caregivers concluded that, to be more involved in the decision-making process, they would need more detailed information about the service user’s health status, better explanations of treatment tactics, and descriptions of medications and their side effects.

Healthcare professionals

Healthcare professionals asked for more training. Training professionals in decision making in psychiatric practice means providing them with the latest theoretical and practical information about service user autonomy and decision making in psychiatry. Specialists expressed a desire to attend informative activities (courses, seminars) that would facilitate a deeper understanding of the concept of service user decision making and enable its practical application.

#### 3.4.2. Other Help and Support

Service users

Service users desired more choice. Several service users thought that they should be trusted more and allowed to make more independent choices about their healthcare. For example, they could choose from the treatment options offered, decide between treatment in a hospital and at home, or select complementary therapies (psychologists, art therapy, etc.).

Service users also desired greater involvement from healthcare professionals in their care. Three service users felt that the more intensive involvement of general practitioners in their care would be highly desirable. Some service users indicated that they would like to receive more attention from HCPs, i.e., to have HCPs show interest in the service user’s daily lives and their goals, instead of asking short, standard questions and prescribing medication.

Family caregivers

Caregivers wished to be seen as reliable partners in the service user’s healthcare. Caregivers thought that they could provide objective information about the service user’s health and behavior based on their daily observations.

In addition to informational support, caregivers desired the greater availability of professionals, easier access to specialists in acute cases, the better exchange of information between professionals and the caregiver, longer and more informative consultations, more openness, more friendliness and compassion from professionals, more attention, and greater consideration of caregivers’ observations about the service user. Caregivers wished to avoid being blamed for the service user’s mental health problems.

Caregivers stressed the need for a more empathetic attitude from other services and institutions, such as emergency services, police, social services, and the courts. Finally, caregivers also expressed the need for additional help, such as support groups (onsite or online), psychological help, and informative seminars specifically designed for caregivers:

*“As a relative [and caregiver], I want to know and understand more. And I always ask why there are no special support groups for relatives who are faced with such patients.”*

*[P2_2]*

Healthcare professionals

One of the cornerstones mentioned was the implementation of the concept of service user decision making in psychiatric care in Latvia. By this, psychiatrists understood, firstly, the need for the thorough acceptance of the concept of service user decision making and, secondly, the importance of developing a unified approach to service user decision making in psychiatry and law.

## 4. Discussion

This paper explores the perspectives of service users, caregivers, and healthcare professionals to identify factors that facilitate or hinder decision making in psychiatric care. By incorporating this triadic viewpoint, the study offers a more comprehensive and nuanced understanding of the decision-making process and its underlying determinants [25]. This viewpoint enables the more extensive and varied justification of the recommendations. Implementing these recommendations would facilitate the fulfillment of both common interests and group-specific roles.

The experiences and concerns of these three stakeholder groups often overlap, although their roles shape differing viewpoints. The study highlights the complexity of psychiatric decision making, which is influenced by legal, personal, and institutional factors. Legal barriers refer to limitations in legislation, as well as regulatory frameworks introduced by hospitals. These frameworks restrict autonomy or informed consent rights. They also fail to provide regulations for meaningful participation. Furthermore, they do not provide norms for the proper implementation of shared and supported decision making. Personal barriers include impairments related to symptoms and other individual factors that affect one’s ability to understand, reason, or communicate throughout the decision-making process. Institutional barriers involve factors such as paternalistic practices, the insufficient training of professionals in decision-making matters, and systemic shortcomings in the availability of and accessibility to healthcare, such as long waiting times or limited access to information. While these categories may overlap, distinguishing between them helps to clarify the specific mechanisms that either support or hinder decision making, as well as the ones that could be used to minimize barriers. The barriers identified within this study reflect the perspectives of other research seeking to describe and define these issues at all levels of the healthcare system—from personal to institutional [46].

### 4.1. Legal Factors for Decision Making in Psychiatry in Latvia

Since 2010, the Patient Rights Law (PRL) has outlined individuals’ rights in healthcare, but psychiatric care, especially inpatient care, remains primarily governed by the Medical Treatment Law (MTL) [4,6]. Article 67(2) of the MTL mandates written consent for hospital admission but does not require informed consent for treatment. Psychiatrists are required to make treatment decisions. Additionally, Article 68(1) permits involuntary treatment that is mandated by a doctors’ council and approved by a court. These legal regulations reinforce substituted decision making.

The application of these provisions in Latvian hospitals was documented by the European Committee for the Prevention of Torture and Inhuman Degrading Treatment or Punishment (CPT) in 2022 [47], as follows:

*“At hospitals […], newly arrived patients were asked upon admission to sign in two places a form of ‘consent to hospitalisation and treatment’, consenting, respectively, to placement and to medical treatment. Hence, by consenting to their treatment at the very outset of hospitalisation—before the clinical indications for a particular form of treatment could possibly be established, patients gave a blanket consent to undergo any treatment regarded as necessary by the treating doctor […]. Thus, it is clear that the consent to treatment given by patients upon admission could not be considered to be an ‘informed consent.’”*

Furthermore,

*“It was clear […] that many of these patients were not at all capable of giving an informed consent to their hospitalisation (or medical treatment) because of severe cognitive deficiencies […].”*

Our data show that service users value being treated as partners in care. They also emphasize the importance of receiving sufficient and detailed information to make decisions. Positive developments are presented by healthcare professionals, who noted improvements in practice and acknowledged the need for and value of user participation.

However, all groups raised concerns about decision making during periods of diminished capacity, whether due to acute symptoms or chronic impairment. The reasons for this are multidimensional. There is a clear legal deficiency: legal regulations in Latvia for persons with temporary or long-term limited capacity are insufficient for autonomy-based decision making. Article 7(1) of the PRL allows decisions by a legal representative or close family when capacity is impaired, but Latvia lacks rules and standardized protocols for the assessment of capacity and the provision of tailored decision-making support. Additionally, there are no legal provisions for the issuance or application of advance directives. This results in frequent errors—such as blanket consent obtained from individuals unable to comprehend or act on it or substituted decisions in cases where appropriate support could enable individuals to make their own decisions.

Service users’ views on how to ensure decision making in such situations varied. Some called for support or the option to delegate decisions to trusted individuals—a right granted under the PRL Article 6(6), as it provides for the right of a patient to authorize another to make decisions. To implement this, service users need to be informed and supported in issuing and registering these authorizations. However, this does not apply to psychiatric inpatient care. Although state-supported decision assistance services exist for people with disabilities [48] in Latvia, their use is currently restricted to outpatient settings. The Cabinet Regulation rules on the provision of assistance in healthcare but limits this assistance up to the doctor’s office door [49]. This limitation is unexplained in regulatory documents.

While service users supported the delegation of decisions to professionals during crises, others raised concerns that this shift could create tensions with user autonomy. Feelings of vulnerability and betrayal were common, while caregivers described emotional distress and the ethical burden of making coercive decisions. Healthcare professionals and caregivers alike raised concerns about the potential misuse of substituted decision making, including fears of manipulation or coercion, particularly as healthcare professionals were also aware of caregivers’ possible hidden motives.

Healthcare professionals suggested that assessing service users’ capacity would allow for more effective treatment planning and the choice of cooperation modes. When assessing their decision-making capacity, psychiatrists may take into account the service user’s interests and whether their choices align with their well-being [43].

A prominent legal barrier is the conflict between confidentiality and family involvement. Caregivers reported exclusion from key decisions due to strict privacy regulations, while healthcare professionals expressed uncertainty about what could be shared without violating confidentiality. This reflects Ní Shé et al. [50], who argue that legal frameworks must better balance service user autonomy and family involvement. We recommend acting in accordance with the PRL Art. 10(2), which requires one to seek the consent of the service user before disclosing their personal information to family caregivers.

Both service users and caregivers expressed doubts about the safety and confidentiality of personal information. In Latvia, it is common for information to be disclosed to service users in the presence of others, despite this being prohibited by law (PRL, Art. 5(7)). These concerns are further compounded by confusion over access rights and fears that any party could violate service users’ right to privacy.

To protect autonomy and enhance the rights of service users, amendments to legislation providing free and informed consent rights within the shared decision-making approach in every healthcare situation are required. Regulations that provide for supported decision making should be put in place. Substituted decision making should be strictly reduced to cases in which a person is objectively unable to make decisions.

### 4.2. Personal Factors That Facilitate or Impede Decision Making 

Personal dynamics significantly shape decision making. Service users highlighted the importance of psychiatrists who foster trust, communicate clearly, and respect their experiences. Negative interpersonal dynamics across all groups were cited as barriers to shared decision making, while trust in clinicians reduced uncertainty and improved outcomes. These findings align with the existing literature that emphasizes the role of trust and transparency in fostering service user autonomy and adherence to treatment plans [11,51].

Cognitive symptoms, acute episodes, and medication side effects were commonly reported to impair decision making. During severe illness, participants supported temporary delegation to caregivers or psychiatrists. These findings align with research suggesting that psychotic symptoms impair appreciation, reasoning, and understanding, making supported decision making essential [52].

This study also underscores the benefits of collaborative or shared decision making involving service users, caregivers, and healthcare providers. Service users’ openness with their doctors and relatives serves as a facilitating factor. This approach leads to personalized care plans and better outcomes [53]. It empowers service users and enables caregivers to offer valuable insights based on their close observations of the service user’s condition. It also enhances caregivers’ ability to monitor changes in the service user’s health status and supports their adherence to healthcare providers’ recommendations throughout the treatment process.

The personal skills of healthcare professionals—such as the ability to listen and truly hear the service user; genuine interest in the service user’s feelings, thoughts, opinions, and quality of life; the professional knowledge of psychiatrists; and the ability to communicate clearly and understandably—were greatly appreciated. When service users and caregivers receive clear and timely information about treatment options, potential side effects, and prognosis, they feel better equipped to participate actively in decision-making processes. This is especially important at the beginning of an illness, when service users often report uncertainty and a lack of knowledge about their diagnosis and its implications for their quality of life. Healthcare professionals play a vital role in service user education, tailoring information to individual needs [53,54].

Caregivers reported uncertainty about how to act and what the best course of action would be in various situations, along with difficulties in managing personal factors such as confusion, doubt, self-recrimination, and feelings of helplessness. They also described emotional challenges—including guilt, despair, anger, pity, and doubt—as well as feelings of relief and even a sense of victory when they were forced to make or enforce coercive decisions. These tensions and emotional responses have been reported in other studies as well [55,56].

### 4.3. Institutional Factors That Facilitate or Impede Decision Making

Participants from all groups identified institutional factors that shaped decision making. The lack of accessible, personalized information was seen as a major barrier, underscoring the importance of providing accurate, user-friendly content about mental illness, treatment options, and available support. Improved access to information, through digital tools, peer support groups, and other channels, was suggested as a key enabler of autonomy and responsibility.

The issue of long waiting times for outpatient consultations in Latvia remains a persistent problem [57,58]. It is, therefore, not surprising that participants emphasized the importance of regular and timely access to healthcare and psychiatric consultations.

Consultations were often described as rushed or too brief. Both service users and healthcare providers suggested that appointments should be longer and better tailored to the specific needs of each service user.

Our findings indicate that paternalistic practices—an institutional factor—remain prevalent in psychiatric care. Many psychiatrists believed that service users with severe psychiatric disorders were incapable of making rational healthcare decisions, leading them to override service user preferences in favor of what they deemed clinically necessary [59]. Moreover, decision making was often influenced by the psychiatrist’s personal beliefs and institutional culture, with inpatient settings being less autonomy-focused than outpatient ones. This reinforces previous research showing that hospitalized service users often experience reduced autonomy due to systemic constraints and paternalistic attitudes [50].

The findings on SDM and its positive benefits are well known from numerous scientific publications and good practice reports. However, these findings have not gained traction in Latvia, so the implementation of this approach is not taking place. This makes Latvia’s situation significantly different from clinical settings in other countries where SDM is already implemented and practiced at the clinic level, such as in the German Share to Care program [60] or in the Netherlands [61].

This study highlights how certain supportive elements—like strong relationships built on trust, open communication, and the active involvement of caregivers—can ease some of the legal and cognitive challenges in psychiatric care. When service users feel that their voices are genuinely heard and respected by their doctors, they are more likely to take part in decisions about their treatment, even when they are struggling with severe symptoms. Relationship barriers—fear and distrust for both service users and clinicians—are well reported [33]. Therefore, these factors should be considered when implementing new approaches in practice.

Caregivers, too, play a key role in supporting a service user. The survey participants stressed the importance of recognizing caregivers as reliable partners in healthcare. When they are well informed and emotionally supported, they can help to steer care away from coercive decisions and toward more collaborative approaches. This suggests that empowering facilitators can transform some barriers into opportunities for enhanced autonomy and care quality. Suggestions included creating support groups, individual and onsite consultations with medical staff, psychological support, and targeted informational events (seminars, workshops, etc.) that could assist and enhance their daily interactions with service users. Healthcare professionals proposed the development of professional caregiving services to support families in their roles.

In summary, this study sheds light on the specific legal and cultural challenges faced in psychiatric care in Latvia, while also contributing to the broader global conversation on ethical and inclusive approaches to decision making in mental health. The findings align with global trends, suggesting that, despite its national focus, this study reflects wider international developments in mental health policy and practice. Addressing these barriers through thoughtful, evidence-based strategies has the potential to bridge the gap between policy intentions and the lived experiences of service users, ultimately promoting both autonomy and quality of care. Expanding our understanding of the needs and interactions between healthcare professionals, caregivers, and service users can further enhance care not only in Latvia but worldwide. The strong international consensus on human rights continues to shift the paradigm in medicine toward greater respect for autonomy and shared decision making. This study offers a broad, multidimensional perspective that supports this ongoing shift—one that ultimately serves those who need our care the most.

We identified two limitations in this study. The first arises from the relatively small number of participants in each study group. Although small samples are typical in qualitative research [62], we initially aimed to include a larger number of participants. However, possibly due to the sensitivity of the topic or other contextual factors, individuals were reluctant to participate. A larger sample size may have allowed for a broader range of perceptions and experiences. The second limitation concerns the study’s cross-sectional design, which precluded our ability to observe changes over time. As a result, we could not follow the development and progression of the decision-making process. Nonetheless, the findings of this study indicate that decision making is an evolving and dynamic process. It may be beneficial to consider conducting a qualitative longitudinal study with a larger sample size to observe how patients’ perceptions and caregivers’ experiences of decision making change over time as care continues. Researching ways to improve existing methods and tools to assess patients’ decision-making capacity, as well as developing new ones, is suggested. Additionally, researching capacity-supporting strategies is recommended.

## 5. Conclusions

This paper examined the perspectives of service users, caregivers, and healthcare professionals to identify factors that facilitate or hinder decision making in psychiatric care. It contributes to a deeper understanding of how decision-making processes in psychiatry are experienced across all three groups within Latvian healthcare institutions. The findings highlight legal, personal, and institutional aspects of these processes and identify several preconditions that influence their feasibility and quality.

Participants acknowledged both the benefits and risks of autonomous decision making. They reported various challenges encountered in practice and suggested ways to improve both systemic and individual mechanisms that are crucial to the quality of decision making.

To support a high-quality decision-making process, which is essential for better health outcomes, the authors propose several recommendations. First, the doctor’s legal right to determine treatment in hospital settings, as outlined in the Medical Treatment Law (MTL), currently enables a paternalistic approach, with limited requirements to uphold the patient’s right to self-determination. The Latvian regulations in this area are comparable to those in many other countries, where mental health laws still restrict the right to free and informed consent for individuals with mental health conditions and psychosocial disabilities, favoring substitute decision making [1]. In order to meet its obligations under the UN Convention on the Rights of Persons with Disabilities, Latvia must consider comprehensive revisions to its mental health legislation. These changes should ensure that the rights of service users are respected, that treatment is provided based on free and informed consent, and that decision-making processes adhere to the principles of a shared decision-making approach.

A human rights-based approach to mental health rejects the segregation of patient rights through specific ‘mental health laws’ [1]. Therefore, the MTL’s provisions on mental illness should be repealed so that all patients’ rights are governed uniformly under the Patient Rights Law.

Secondly, personal factors must be consistently considered and appropriately addressed in the decision-making process. Healthcare providers should assess the capabilities of both service users and their caregivers, promoting factors that support decision making—such as psychoeducation, improved health literacy, and access to peer support. Barriers should be reduced through tailored, sensitive measures, including providing calming environments and allowing additional time for decision making. We recommend evidence-based approaches such as de-escalation techniques and the Open Dialogue model, alongside technological solutions like remote consultations with specialists or support persons.

The ability of caregivers and healthcare professionals to facilitate sound decision making is influenced by both personal and systemic factors. Caregivers’ capacity to offer effective support can be strengthened through targeted interventions that reduce stress and mitigate perceived risks. Dedicated support measures for caregivers would further enhance their ability to engage in shared decision making. For healthcare professionals, their personal capacity can be improved through occupational risk reduction strategies, including burnout prevention, individual supervision, and targeted training. Training should focus on assessing and supporting the decision-making capacity, effective communication, and the selection and implementation of support measures for individuals with mental health conditions and reduced autonomy.

Thirdly, as with personal factors, institutional elements can either promote or hinder decision making. The study data and participant suggestions point to key institutional components that require improvement. Chief among these are the availability, accessibility, and reliability of information about mental health treatment. Institutional factors should be evaluated for their influence—positive or negative—on decision making. Solutions should include reviewing the clarity and appropriateness of informational materials for service users and revising content that is overly complex, misleading, or scientifically unsupported.

In order to effect positive change, it is crucial to engage in a public and professional dialog based on this study and others like it. Raising awareness of the challenges faced by service users in psychiatric care at both societal and institutional levels will enable targeted efforts to address them. Ultimately, such efforts can improve decision-making practices, enhance service user autonomy, and promote truly person-centered care.

## Figures and Tables

**Table 1:** Inclusion criteria for the participants.

Participants	Inclusion Criteria
Service Users	(1) aged 18 years or older; (2) having a mental illness diagnosed by a psychiatrist for at least 2 years (except impaired consciousness, dementia, an acute psychotic state, psychomotor agitation, disorganized thinking, moderate/severe mental retardation, severe depression/manic illness, moderate/pronounced cognitive decline, under the influence of neuroleptics or tranquillisers, high anxiety levels, and severe pain); (3) a history of at least one treatment in the hospital as an inpatient; (4) interest in participating in the study; (5) the ability to talk about their experiences.
Family caregivers	(1) aged 18 years or older; (2) care of an adult with a mental illness for at least 2 years; (3) related to the patient by blood or marriage; (4) not a professional caregiver; (5) provides care at no charge; (6) interest in participating in the study; (7) the ability to talk about their experiences.
Healthcare professionals	(1) professionals (psychiatrists and nurses) working in mental healthcare; (2) at least 3 years of practice in mental healthcare; (3) interest in participating in the study; (4) the ability to talk about their experiences.

**Table 2:** Characteristics of the research participants.

Variable		Service Users (N = 8)	Family Caregivers (N = 6)	Healthcare Professionals (N = 7)
Gender	Female	7	3	4
	Male	1	3	3
Age	20–30	3	-	-
	31–40	3	-	5
	41–50	1	3	1
	51–60	1	2	1
	60+	-	1	-
Service user’s primary diagnosis/Primary diagnosis of the service user being cared for by the caregiver	Bipolar disorder	1	1	
	Schizophrenia	2	3	
	Depressive disorder	2	2	
	Schizotypal personality disorder	1	-	
	Unknown	2	-	
Caregiver relationship with the service user	Parent		3	
	Son or daughter		2	
	Husband or wife		1	
Duration of disease (years)	2–5	1		
	6–10	4		
	11+	3		
Years of working in psychiatry	3–10			4
	11–15			2
	16+			1
Setting	Inpatient			2
	Outpatient			4
	Forensic			1
Specialization of healthcare professional	Physician (Psychiatrist)			5
	Nurse (in psychiatry)			2

## Data Availability

The quotations in the present paper/study are based on transcribed interviews that contained sensitive/personal information; therefore, the data cannot be made publicly available. The anonymized data that support the findings of this study are available from the corresponding author upon reasonable request.

## Supplementary Materials

The following supporting information can be downloaded at: https://www.mdpi.com/article/10.3390/healthcare13121416/s1, Table S1: Topics covered in the interviews, Table S2: Categories, sub-categories and main codes of the study.
