# Supplementary material for: Triadic Perspectives on Decision Making in Psychiatry: A Qualitative Study on Service Users, Caregivers and Healthcare Professionals in Latvia
Source: Healthcare (Basel). 2025 Jun 13;13(12):1416. doi: 10.3390/healthcare13121416 (PMC12192966; doi:10.3390/healthcare13121416)
Supplement: Supplementary file 1 [file healthcare-13-01416-s001.zip › healthcare-3604317-supplementary.pdf]

healthcare

MDPI

Table S1. Topics covered in the interviews.

Service users	· Experience of being diagnosed with a mental illness<br>· General views on decision making in psychiatric care<br>· Practical experience with decision-making in psychiatric care<br>· Expectations for the preferred decision-making model
Caregivers	· Perceptions of decision-making issues in mental health<br>· Practical experience in decision making while caring for a person with mental illness<br>· Reflections on possible barriers and challenges of decision-making<br>· Preferences related to decision-making<br>· Recommendations for improving the decision-making process
Healthcare professionals	· Invitation to describe the principle of service user autonomy in psychiatric care during the last 5–10 years in Latvia<br>· General opinion on decision-making issues<br>· Factors affecting the decision-making process<br>· Practical experience regarding decision-making<br>· Improvements to strengthen the decision-making process in psychiatric care

Table S2. Categories, sub-categories and main codes of the study.

Categories	Subcategories	Main codes	Perspective
**1. The antecedents for decision-making**	1.1 Factors related to mental healthcare	· Access to HCPs<br>· Appropriate duration of consultation<br>· Qualities of HCPs<br>· Provision of information	*Service users*
		· Unavailability of service user information<br>· Service users' right to be informed	*Family caregivers*
		· Strengthening service users' rights in mental healthcare<br>· Information available to service users<br>· HCP's personality and place of practice	*Healthcare professionals*
	1.2 Factors related to service user	· Their attitude<br>· Having a reliable support person<br>· Mental health situation	*Service users*
		· Service user's ability to cooperate<br>· Service user's current mental health situation	*Family caregivers*
		· Active service user<br>· Service user's mental health condition	*Healthcare professionals*
**2. Challenging moments in the decision-making practice**	**-**	· Decision-making at the onset of the disease<br>· Outpatient facility versus hospital<br>· Conflicting information	*Service users'*
		· The onset of the service user's illness and the caregiver's tough decision	*Family caregivers'*

		· Forced to take part in coercion<br>· Disagreement on what constitutes coercion<br>· Who is the main decision maker regarding service user healthcare?<br>· Justification of coercion by caregivers	
		· Between service user and caregiver on disclosure of information<br>· Service user's relationship with the caregiver<br>· Difficulties in assessing a service user's decision-making capacity<br>· Deciding that you must decide for the service user	*Healthcare professionals'*
**3. The consequences and side effects of decision-making**	3.1 Benefits	· Benefits related with medical treatment<br>· Felt more safe and secure<br>· Sense of control<br>· Felt important<br>· Felt empowered	*Service users'*
		· Reduced caregivers' responsibility<br>· Increased service user motivation	*Family caregivers*
		· Benefit from an assessment of the service user's decision-making capacity<br>· Choose a food	*Healthcare professionals'*
	3.2 Risks	· Wrong, dangerous, harmful decisions	*Service users'*
		· Risks of service user abuse	*Family caregivers*
		· The true intentions of significant Others	*Healthcare professionals' perspective*
	3.3 Burden	· Additional burden, especially if you feel ill<br>· Little interest in health topics	*Service users'*
		· Additional burden on caregiver daily responsibilities	*Family caregivers'*
		· Additional resources	*Healthcare professionals*
**4. Recommendations for better decision-making**	4.1 Informational support	· More information	*Service users*
		· More information	*Family caregivers'*

		· Training professionals in decision-making in psychiatric practice	*Healthcare professionals'*
	4.2 Other help and support	· More choices<br>· More involvement of HCP in service user care	*Service users'*
		· Caregivers as reliable partners<br>· More support from the HCP<br>· More empathy from other services and institutions<br>· Additional help for caregivers	*Family caregivers*
		· Implementation of the concept of service user decision-making in psychiatric care in Latvia	*Healthcare professionals*
